# Nanoparticle-Loaded Polarized-Macrophages for Enhanced Tumor Targeting and Cell-Chemotherapy

**DOI:** 10.1007/s40820-020-00531-0

**Published:** 2020-10-27

**Authors:** Teng Hou, Tianqi Wang, Weiwei Mu, Rui Yang, Shuang Liang, Zipeng Zhang, Shunli Fu, Tong Gao, Yongjun Liu, Na Zhang

**Affiliations:** Department of Pharmaceutics, Key Laboratory of Chemical Biology (Ministry of Education), School of Pharmaceutical Sciences, Cheeloo College of Medicine, Shandong University, 44 Wenhuaxi Road, Jinan, 250012 People’s Republic of China

**Keywords:** Polarized-macrophages, Cell therapy, Cell-mediated drug delivery, Chemotherapy, Lipid nanoparticles

## Abstract

Cell therapy is a promising strategy for cancer therapy. However, its therapeutic efficiency remains limited due to the complex and immunosuppressive nature of tumor microenvironments. In this study, the “cell-chemotherapy” strategy was presented to enhance antitumor efficacy. M1-type macrophages, which are therapeutic immune cells with both of immunotherapeutic ability and targeting ability, carried sorafenib (SF)-loaded lipid nanoparticles (M1/SLNPs) were developed. M1-type macrophages were used both as therapeutic tool to provide immunotherapy and as delivery vessel to target deliver SF to tumor tissues for chemotherapy simultaneously. M1-type macrophages were obtained by polarizing macrophages using lipopolysaccharide, and M1/SLNPs were obtained by incubating M1-type macrophages with SLNP. Tumor accumulation of M1/SLNP was increased compared with SLNP (*p* < 0.01), which proved M1/SLNP could enhance tumor targeting of SF. An increased ratio of M1-type macrophages to M2-type macrophages, and the CD3^+^CD4^+^ T cells and CD3^+^CD8^+^ T cell quantities in tumor tissues after treatment with M1/SLNP indicated M1/SLNP could relieve the immunosuppressive tumor microenvironments. The tumor volumes in the M1/SLNP group were significantly smaller than those in the SLNP group (*p* < 0.01), indicating M1/SLNP exhibited enhanced antitumor efficacy. Consequently, M1/SLNP showed great potential as a novel cell-chemotherapeutic strategy combining both cell therapy and targeting chemotherapy.

**Electronic supplementary material:**

The online version of this article (10.1007/s40820-020-00531-0) contains supplementary material, which is available to authorized users.

## Highlights

- A polarized-macrophages-based drug delivery system (M1/SLNP) was presented for the cell-chemotherapy of cancer.
- Polarized-macrophages were used both as therapeutic tool to provide immunotherapy and as delivery vessel to target deliver chemotherapeutic drugs to tumor tissues for chemotherapy simultaneously.
- M1/SLNP was a multifunctional delivery system with simple structure, excellent safety, and without complex synthesis process.

**Electronic supplementary material:**

The online version of this article (10.1007/s40820-020-00531-0) contains supplementary material, which is available to authorized users.

## Graphical abstract

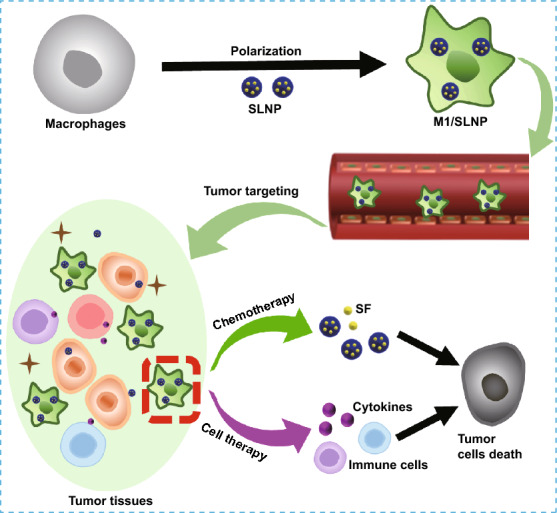

## Introduction

Cell therapy has emerged as a novel immunotherapeutic approach for cancer treatment, by directly transporting therapeutic immune cells, such as T cells, NK cells, and macrophages, to eliminate cancer cells [1–3]. Compared with traditional molecule drugs, cells with exquisite sensitivity and specificity can sense diverse signals, move to specific sites in the body, and execute complex response behaviors [4]. Based on these characteristics, cell therapy has the advantages of higher specificity and lower side effects [5]. Many clinical trials underway have highlighted the benefits of using cells as therapeutic agents [6]. Kymriah, a cell-based gene therapy, was the first chimeric antigen receptor T cells (CAR-T) therapy approved by the FDA in 2017 [7]. Despite these encouraging approaches, the therapeutic efficiency of cell therapy remains limited due to the complex and immunosuppressive tumor microenvironments [8, 9]. Some studies have developed smart generations of cell therapy strategies to circumvent these limitations, such as the fourth-generation CAR-T was engineered to express pro-inflammatory cytokines, combining CAR-T therapy with immune checkpoint inhibitors and combining CAR-T therapy with vaccines [10–12]. However, challenges such as complex production processes and high production costs have slowed down their development rate [9].

Herein, we hypothesized a “cell-chemotherapy” strategy using drug-loaded therapeutic cells to enhance the antitumor efficacy of therapeutic cells. On the one hand, the cells were used as a therapeutic tool to kill cancer cells or provide immunotherapy; on the other hand, the cell was used as a delivery vessel to target drugs to tumor tissues and achieved chemotherapy. This strategy showed unique synergistic advantages: Therapeutic cells could trigger the antitumor immune response and then kill tumor cells. Also, therapeutic cells could enhance the tumor targeting of chemotherapeutic drugs; chemotherapeutic drugs could directly kill tumor cells and improve the sensitivity of tumor cells to cell therapy.

Macrophages are the major tumor-infiltrating immune cells population with a critical role in regulating tumor progression, induced by the tumor microenvironments to differentiate into M1-type macrophages (M1) and M2-type macrophages (M2) [13–15]. M1-type macrophages secreting immunogenic cytokines, such as IL-12 and TNF-a, improve the immune response that exerts inhibitory effects on tumor growth, and M2-type macrophages secreting immunosuppressive cytokines, such as IL-10 and TGF-β, impair antitumor immunity to enhance tumor growth [16, 17]. Macrophage-based cell therapy strategies have been widely developed [3, 18, 19]. For example, Zhang et al. designed a chimeric antigen receptor-modified macrophage that significantly inhibited tumor growth after intravenous injection [20]. Apart from as one kind of the important immune cells involved in cancer immunity, macrophages are one of the most abundant types of circulating cells in body [14, 21]. Compelling evidence has shown that macrophages can be recruited to tumor tissues by some chemokines, such as CCL2 [22, 23]. Moreover, macrophages are major phagocytes with innate phagocytotic capability [24]. Based on their tumor targeting and phagocytotic capability, macrophages might be an ideal tool for tumor-targeted drug delivery [25, 26]. Researchers have developed potential strategies using macrophages to deliver small drug molecules or drug-loaded nanoparticles effectively [27–30]. For example, Fu et al. constructed a biomimetic delivery system with promising antitumor efficacy using a mouse macrophage-like cell (RAW264.7) to deliver doxorubicin [31]. An et al. designed a RAW264.7-mediated small gold nanorods delivery system achieving high drug accumulation in tumor sites [32]. Consequently, macrophages are expected to be a promising type of cell used for the cell-based treatment and drug delivery system.

The method for loading chemotherapeutic drugs into macrophages is another concern in cell-chemotherapy. The ideal method needs to have high drug-loading and appropriate drug-release profiles with low toxicity for macrophages, and does not affect the functions of macrophages or drugs [33]. Nanoparticles provided a protective approach for macrophages. Drug-loaded nanoparticles were formed by loading chemotherapeutic drugs into nanoparticles and then loaded into macrophages, preventing drug damage to the cells [34, 35]. Lipid nanoparticles (LNPs) with favorable biocompatibility and excellent safety have been widely used as drug delivery vessels [36–38]. Patisiran, a drug delivered by LNP, was approved by the FDA in 2018 [39]. Using LNP to encapsulate drugs, on the one hand, avoided the damage of drugs to macrophages, and on the other hand, avoided the damage of carrier materials to macrophages. Meanwhile, the preparation method was simple and without complex synthesis process.

**Scheme 1:**
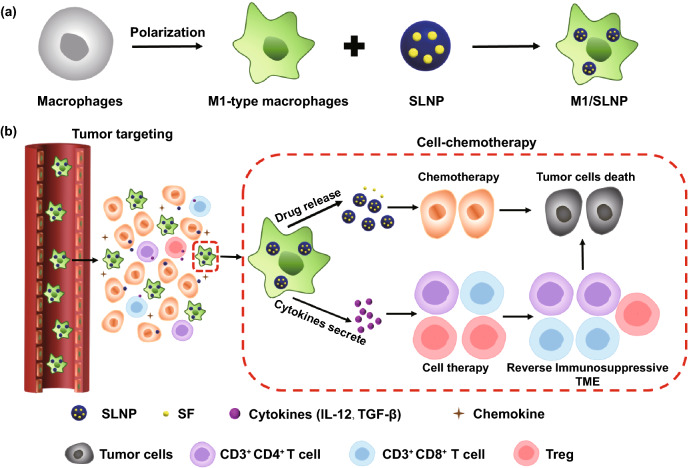
**a** Preparation of M1/SLNP. **b** Schematic illustration of M1/SLNP for tumor targeting delivery to enhance the therapeutic efficiency of HCC, in which dual functional M1-type macrophages as targeting delivery vessel and therapeutic tool

In this study, a polarized macrophage-based treatment and drug delivery system involving M1-type macrophages carrying sorafenib (SF)-loaded lipid nanoparticles (M1/SLNP) were designed for the cell-chemotherapy of hepatocellular carcinoma (HCC). SF is a multityrosine kinase inhibitor that blocks tumor cell proliferation and the first-line drug approved by the FDA in 2007 for the treatment of HCC [40–42]. SF could significantly prolong the survival time in advanced HCC patients and showed high inhibition on HCC cell lines such as Hepa1-6 cell line and HepG2 cell line [43–47]. Macrophages were polarized into M1-type macrophages with immunotherapeutic efficiency. The CCL2 required for recruitment of macrophages was overexpressed in HCC, and macrophages could be recruited to the tumor tissues effectively [48]. M1/SLNP showed unique advantages: M1-type macrophages were used as an immunotherapeutic tool to involve in cell therapy to modulate the tumor immune microenvironment from immunosuppressive state to immune activated state; M1-type macrophages were also utilized as a chemotherapeutic drugs delivery tool to deliver SF and enhanced the tumor targeting of SF; SLNPs prevent the toxic effects of SF on the M1-type macrophages and M1-type macrophages could maintain the functions; SLNP with small particle size released from M1/SLNP exhibited deep tumor-penetrating ability; M1/SLNP was a multifunctional delivery system with simple structure, excellent safety and without complex synthesis process. As shown in Scheme 1, M1/SLNPs were recruited to the tumor tissues by the homing effect of macrophages firstly; then, SLNPs were released from M1/SLNP to display the chemotherapy effects. SLNPs were expected promote the deep infiltration of tumor. Meanwhile, M1-type macrophages acted as the immunotherapeutic tool, secreting immunogenic cytokines, and relieve the tumor immunosuppressive microenvironments.

In this study, M1/SLNP was prepared successfully. The morphology and particle size of SLNP were investigated. The phenotype, release properties, and deep tumor-penetrating ability of M1/SLNP were studied. In addition, the tumor targeting ability of M1/SLNP was proved in vitro and in vivo. Moreover, the relieved immunosuppressive tumor microenvironments were evaluated by in vivo immunization study. The antitumor efficacy was evaluated in vitro and in vivo. This study provides a potential approach of novel macrophage-based therapy strategy with enhanced antitumor activity.

## Experimental Section

### Materials

SF was provided by Shanghai Biochempartner Co., Ltd. (Shanghai, China). Injectable soya lecithin was provided by Shanghai Taiwan Pharmaceutical Co., Ltd. (Shanghai, China). Coumarin-6 (C6) was bought from Aladdin Chemical Co., Ltd. (Shanghai, RPC). DSPE-rhodamine B was purchased from Ruixi Biological Technology Co., Ltd (Xi’an, China). Methylthiazol tetrazolium (MTT) were purchased from Sigma-Aldrich (US). APC anti-mouse CD3, FITC anti-mouse CD4, PE anti-mouse CD8a, PE anti-mouse CD25, and Alexa Fluor® 647 anti-mouse FOXP3 were purchased from eBioscience. Alexa Fluor ® 488 anti-mouse CD86, PerCP/Cy5.5 anti-mouse F4/80, and APC anti-mouse CD206 were bought from eBioscience. Mouse IL-12p70 Elisa kit, Mouse TGF-β1 Elisa kit, Mouse IL-10 Elisa kit, and Mouse TNF-α ELISA kit were purchased from DAKEWE. All other reagents were of analytical grade and obtained commercially.

### Cell Culture

Macrophages (RAW264.7 cells, a murine macrophage cell) and Hepa1-6 cells were cultured in DMEM medium supplemented with 10% fetal bovine serum. All cells were cultured in a 37 °C incubator with 5% CO_2_.

### Animals

Female C57BL/6 mice (6–8 weeks old) and female Kunming mice (6–8 weeks old) were provided by the Medical Animal Test Center of Shandong University (Jinan, China). All experiments complied with the requirements of the Animal Management Rules of PRC (document No. 55, 2001) and of the Laboratory Animal Ethical and Welfare Committee of Cheeloo College of Medicine, Shandong University.

### Preparation of SLNP

SLNPs were prepared by nanoprecipitation methods. SF was dissolved in 1 mL methyl alcohol. Soya lecithin was dissolved in Tween-80 aqueous solution (1.5%, w/v). The organic phase was added to Tween-80 aqueous solution under constant mechanical agitation using a microsyringe pump (KD Scientific, MA, USA). SLNPs were obtained after methyl alcohol evaporation. The optimal formulations were determined by single factor studies. SF/soya lecithin mass ratio and the soya lecithin concentration were investigated. For the preparation of C6-LNP and Cy5.5-LNP, SF was replaced with C6 at a concentration of 600 μg mL^−1^ and Cy5.5 at a concentration of 500 μg mL^−1^. The other procedures were similar to that for SLNP.

### Characterization of SLNP

The morphology of SLNP was characterized by transmission electronic microscopy (TEM). The particle sizes and polydispersity index (PDI) of SLNP were determined by a Zetasizer Nano ZS90 (Malvern, UK). SF was quantitatively analyzed using high-performance liquid chromatography (HPLC) (SPD-10Avp Shimadzu pump, LC-10Avp Shimadzu UV–Vis detector). Equation (1) is used to calculate the drug-loading efficiency (DL%) of SLNP:

1 $$\mathrm{DL\%}=\frac{{W}_{\mathrm{drug}}}{{W}_{drug}+{W}_{\mathrm{lsoya lecithin}}}\times 100$$

*W*_drug_ was the drug weight, and *W*_soya lecithin_ was the soya lecithin weight.

### In vitro Release of SF from SLNP

In vitro release of SF from SLNP was conducted by the dialysis bag diffusion method. 1 mL of SLNP (15 μg mL^−1^), SF solution (15 μg mL^−1^, Taxol prescription diluted with release medium) were added into dialysis bags (8 to 14 kDa molecular weight cutoff), respectively. The release media for SLNP and SF solution were pH 7.4 phosphate-buffered saline (PBS) included Tween 80 (1%, w/v) and pH 6.5 PBS included Tween 80 (1%, w/v), respectively. The bags were incubated in 10-mL release medium at 37 °C under horizontal shaking. At the predetermined time points, the release medium was obtained and replaced with fresh medium. The released SF was quantitatively analyzed by HPLC. The experiments were carried out in triplicate.

### Preparation of M/SLNP and M1/SLNP

Macrophages carrying SLNP (M/SLNP) and M1/SLNP were obtained by incubating macrophages and M1-type macrophages with SLNP, respectively. M1-type macrophages were obtained by incubating macrophages with LPS at a concentration of 1 µg mL^−1^ for 24 h. Macrophages and M1-type macrophages (1 × 10^6^ cells mL^−1^) were seeded in a sterile tube and incubated with SLNP (200 µg mL^−1^) for 2 h at 37 °C. Cells are centrifuged to separate from the SLNP solution and then resuspended in PBS to obtain the M/SLNP and M1/SLNP suspension. To get the total amount of drug loading in cells, SF in SLNP before and after incubating with cells was quantitatively measured using HPLC. The optimal formulations for M/SLNP were determined by single factor study. The concentration of SF and incubation time were investigated. The cytotoxicity of SLNP on macrophages was tested by MTT assay. Macrophages were seeded into 96-well plates (5000/well). A series of doses of SLNP at a SF concentration of 50, 100, 200, 300, and 400 μg mL^−1^ were added to the wells and incubated for 1, 2, and 4 h. Then, SLNPs were removed, and DMEM medium supplemented with 10% fetal bovine serum was added to each well. After incubating for 48 h, MTT and DMSO was added. The cell viability was measured by a microplate reader (Model 680, BIO-RAD, CA, USA).

For preparation of macrophages carrying SF (M/SF), macrophages were incubated with SF solution (200 µg mL^−1^) for 2 h at 37 °C. The subsequent procedures were similar to that for M/SLNP. The optimal formulation for M/SF was similar to that for M/SLNP.

### Characterization of M/SLNP and M1/SLNP

The in vitro phenotype of macrophages was evaluated by flow cytometry (FCM) assay. Macrophages (2 × 10^5^ cells) were cultured overnight in a 12-well plate. M1-type macrophages were obtained by incubating macrophages with LPS at a concentration of 1 µg mL^−1^ for 24 h. Subsequently, SLNPs were added and incubated with M1-type macrophages for 2 h to obtain M1/SLNP. M1/SLNPs were marked with PerCP/Cy5.5 anti-mouse F4/80, Alexa Fluor® 488 anti-mouse CD86 and APC anti-mouse CD206, and analyzed by FCM. F4/80^+^CD86^+^ cells were M1-type macrophages, and F4/80^+^CD206^+^ cells were M2-type macrophages. In addition, M1/SLNPs were incubated for 24 h. The level of cytokines in the supernatant secreted from M1-type macrophages was analyzed using the ELISA kit.

Confocal laser scanning microscopy (CLSM) and TEM were used to confirm that the SLNP had been successfully loaded into macrophages and M1-type macrophages. Briefly, macrophages and M1-type macrophages were incubated with C6-LNP (20 µg mL^−1^) for 2 h at 37 °C to obtain macrophages carrying C6-loaded lipid nanoparticles (M/C6-LNPs) and M1-type macrophages carrying C6-loaded lipid nanoparticles (M1/C6-LNPs), respectively. M/C6-LNP and M1/C6-LNP were stained with Alexa Fluor ® 647 anti-mouse F4/80 by incubating with Alexa Fluor ® 647 anti-mouse F4/80 (1.5 μg mL^−1^) for 1 h. After washing with PBS thrice, the double-stained F4/80-M/ C6-LNP and F4/80-M1/ C6-LNP were observed by CLSM. M/SLNP and M1/SLNP were observed using TEM.

The endocytic pathway of SLNP in macrophages was investigated by FCM. Macrophages (2 × 10 ^5^ cells/well) were seeded into 12-well plates and pre-incubated with cytochalasin D (30 mM), genistein (1 μg mL^−1^), or chlorpromazine (10 μg mL^−1^). Then, the cells were incubated with C6 or C6-LNP for 1 h and evaluated using FCM.

### In vitro Release of SF from M/SLNP or M1/SLNP

To evaluate the in vitro release properties of SF from M/SLNP or M1/SLNP, macrophages were cultured overnight in 12-well plates. M1-type macrophages were obtained by incubating macrophages with LPS at a concentration of 1 µg mL^−1^ for 24 h. Macrophages or M1-type macrophages were incubated with SLNP (200 µg mL^−1^) for 2 h at 37 °C and then incubated with fresh DMEM medium supplemented with 10% fetal bovine serum for different periods (0.5, 1, 2, 4, 8, 12, 24, 48, and 72 h). At predetermined time points, the released medium from each well was obtained and the amount of the total released SF in the released medium was determined using HPLC. To clarify that SF would be released from M/SLNP or M1/SLNP as SF or SLNP, the released medium was added into a centrifugal filter device (10 K MWCO), followed by centrifugation (5000 g, 15 min). The released SF in the filtrate was quantitatively analyzed using HPLC. The amount of the released SLNP (*A*_t_ − *A*_f_) was calculated, where *A*_t_ and *A*_f_ are the amount of the total released SF in the released medium and the released SF in the filtrate, respectively. In addition, the released medium after 24 h was taken and observed using TEM.

### Stability of SLNP in M/SLNP or M1/SLNP

CLSM and TEM were used to evaluate the stability of SLNP in M/SLNP or M1/SLNP. Briefly, DSPE-rhodamine B and soya lecithin were dissolved in Tween-80 aqueous solution (1.5%, w/v), and the other procedures were similar to that for C6-LNP. Thus, C6-LNP were obtained, in which LNP were fluorescently labeled by rhodamine B. Macrophages were cultured overnight in 12-well plates. M1-type macrophages were obtained by incubating macrophages with LPS (1 µg mL^−1^) for 24 h. C6-LNPs were added and incubated with macrophages or M1-type macrophages for 2 h. After washing by PBS, the cells were incubated with fresh DMEM medium supplemented with 10% fetal bovine serum for different periods (0, 4, 8, 12, and 24 h). At predetermined time points, the cells were washed with PBS and observed under CLSM. In addition, the cells at 24 h were obtained and visualized under TEM.

### Tumor-Penetrating Ability in vitro

Each well of the 96-well plates was pre-coated with fetal bovine serum-free medium containing sterile agarose. Hepa1-6 cells (5000 cells/well) were seeded into each well and cultured in the medium containing FBS (10%, v:v). The tumor spheroids were allowed to grow at 37 °C for 7 days. Macrophages were cultured overnight in 12-well plates. M1-type macrophages were obtained by incubating macrophages with LPS (1 µg mL^−1^) for 24 h. C6-LNPs were added and incubated with macrophages or M1-type macrophages for 2 h to obtain M/C6-LNP and M1/C6-LNP, respectively. The cells were washed with PBS and then incubated with fresh DMEM medium supplemented with 10% fetal bovine serum for 24 h to obtain the released medium. Subsequently, the tumor spheroids were incubated with free C6, C6-LNP, the released medium from M/C6-LNP or the released medium from M1/C6-LNP for 6 h. The tumor spheroids were washed with PBS. The tumor spheroid images were acquired by CLSM.

### Tumor Targeting Capability in vitro and in vivo

The chemotaxis of M/SLNP and M1/SLNP was investigated using a Transwell migration assay in vitro (Transwell polycarbonate membrane, 8 µm pore size, 6.5 mm diameter and 0.33 cm^2^ membrane surface area, Corning). M/SLNP and M1/SLNP were suspended in DMEM medium and plated in the upper chamber of the Transwell. The lower compartment was filled with fresh DMEM medium or Hepa1-6 conditioned media collected from DMEM medium after culturing Hepa1-6 cells for 48 h. After incubating for 6 h at 37 °C, the cells migrating across the Transwell in the lower chamber were detected under a fluorescence microscope (BX40; Olympus Corporation, Tokyo, Japan).

To investigate the tumor targeting capabilities of M/SLNP and M1/SLNP, macrophages and M1-type macrophages were incubated with C6-LNP (20 µg mL^−1^) for 2 h at 37 °C to obtain M/C6-SLNP and M1/C6-LNP, respectively. Then, DiI staining macrophages and M1-type macrophages were incubated with M/C6-LNP and M1/C6-LNP at 37 °C for 2 h to obtain DiI-M/C6-LNP and DiI-M1/C6-LNP. The Hepa1-6 tumor-bearing C57BL/6 mice were used as animal models, which were established by inoculating subcutaneously 1 × 10^7^ Hepa1-6 cells at the right axillary. When the tumors grew to approximately 200 mm^3^, the mice were intravenously injected with DiI-M/C6-LNP or DiI-M1/C6-LNP (5 × 10^6^ cells/mouse). The mice were sacrificed after 24 h, and the tumors were collected and cryo-sectioned at a thickness of 10 μm. The sections were imaged by CLSM.

The in vivo biodistribution of M/SLNP and M1/SLNP was investigated. Macrophages and M1-type macrophages were incubated with Cy5.5-LNP (30 µg mL^−1^) at 37 °C for 2 h to macrophages carrying Cy5.5-loaded lipid nanoparticles (M/Cy5.5-LNP) and M1-type macrophages carrying Cy5.5-loaded lipid nanoparticles (M1/Cy5.5-LNP), respectively. Considering that the black hair of C57BL/6 mice might affect the fluorescence signal, the Hepa1-6 tumor-bearing Kunming mice were used as animal models, which were established by inoculating subcutaneously 1 × 10^7^ Hepa1-6 cells at the right axillary. When the tumors grew to approximately 200 mm^3^, the mice were intravenously injected with free Cy5.5, Cy5.5-LNP, M/Cy5.5-LNP, or M1/Cy5.5-LNP (1 mg kg^−1^). The mice were anesthetized after 1, 2, 4, 8, 12, and 24 h of injection and observed with the in vivo real-time fluorescence imaging system (IVIS) spectrum (Caliper PerkinElmer, Waltham, MA, USA). For further ex vivo evaluation, the mice were sacrificed at 12 or 24 h, and tumors or organs were obtained. In addition, to investigate the tumor-penetrating ability in vivo, the tumors were obtained and cryo-sectioned at a thickness of 10 μm, and the tumor sections were labeled with DAPI.

### Cellular Uptake Study

Hepa1-6 cells (1 × 10^5^ cells/well) were seeded into 12-well plates. Free C6, C6-LNP, the released medium from M/C6-LNP or the released medium from M1/C6-LNP (100 ng mL^−1^) were added and further incubated for 1 or 2 h. After washing with PBS, the cells were imaged by fluorescence microscope. In addition, to quantifying the cellular uptake, cells treated with free C6, C6-LNP, the released medium from M/C6-LNP and the released medium from M1/C6-LNP were collected and measured using FCM. The preparation of the release medium from M/C6-LNP and the release medium from M1/C6-LNP were the same as described in the “Tumor-penetrating Ability in vitro” part.

### In vitro Cytotoxicity Assay

The cytotoxicity of M1/SLNP in Hepa1-6 cells was investigated by MTT assay. Briefly, Hepa1-6 cells (5000 cells/well) were incubated into 96-well plates. LNP, free SF, SLNP, released medium of M/LNP, released medium of M1/LNP, released medium of M/SLNP, and release medium of M1/SLNP (0.1, 1, 5, 10, and 20 μg mL^−1^) were added and incubated for 48 h. LNP was added at concentration equal to SF in SLNP. Released medium of macrophages (released medium of M) and released medium of M/LNP (released medium of M/LNP) were added at concentration equal to SF released from M/SLNP. Released medium of M1-type macrophages (released medium of M1) and released medium of M1/LNP (released medium of M1/LNP) were added at concentration equal to SF released from M1/SLNP. After incubating for 48 h, MTT and DMSO were added. The cell viability was measured by a microplate reader. The following formula (2) was used to calculate the relative cell viability (%):

2 $$ {\text{Relative cell viability }}\left( \% \right) = \left( {A_{{{\text{sample}}}} /A_{{{\text{control}}}} } \right) \times {1}00\% $$

Macrophages were cultured overnight in 12-well plates. M1-type macrophages were obtained by incubating macrophages with LPS (1 µg mL^−1^) for 24 h. Macrophages or M1-type macrophages were incubated with SLNP (200 µg mL^−1^) for 2 h at 37 °C. After washing by PBS, the cells were incubated with fresh DMEM medium supplemented with 10% fetal bovine serum for 24 h. Released medium of M/SLNP and released medium of M1/SLNP were obtained in the supernatant. The preparation of released medium of M and released medium of M/LNP was similar to that of released medium of M/SLNP. The preparation of released medium of M1 and released medium of M1/LNP was similar to that of released medium of M1/SLNP.

### In vivo Antitumor Efficacy

The antitumor efficacy of M1/SLNP was evaluated using the Hepa1-6 tumor-bearing C57BL/6 mice model. The mice were randomly separated into seven groups (*n* = 6). The mice were intravenously injected with NS, M, M1, free SF, SLNP, M/SLNP, and M1/SLNP (7 mg kg^−1^ SF, about 4 × 10^6^ macrophages or M1-type macrophages) every 4 days for five times. The tumor volume and body weight were measured every other day. At day 19 after the first administration, the mice were sacrificed and tumors were excised, photographed and weighed. The tumor inhibition rate was calculated for different groups, and the following formula (3) was used to calculate the tumor inhibition rate (Ti) for different groups:

3 $$ {\text{Ti(\% )}} = \left( {V_{{\text{s}}} - V_{{\text{i}}} } \right)/V_{{\text{s}}} \times 100 $$

Ti represents the tumor inhibition rate for different groups (M, M1, free SF, SLNP, M/SLNP, and M1/SLNP group, respectively); *V*_s_ represents the mean tumor volume of saline group; *V*_i_ represents the mean tumor volume of different groups (M, M1, free SF, SLNP, M/SLNP, and M1/SLNP group, respectively).

### Immunohistochemistry Evaluation

After the in vivo antitumor efficacy study, major organs (heart, liver, spleen, lung, and kidney) and tumors were obtained and then fixed in 4% paraformaldehyde and embedded in paraffin wax for histological analysis. The sections were stained with hematoxylin and eosin (H&E). In addition, the tumor sections were stained with Ki67 to evaluate the cell proliferation.

### In vivo Immunization Study

The in vivo macrophages phenotype was evaluated by FCM. After the in vivo antitumor efficacy study (on day 19 after the first administration), tumor tissues were obtained, and then, tissues were ground, filtered by a copper network. Following centrifugation (1500 rpm, 10 min), the total cells were collected and counted, staining with PerCP/Cy5.5 anti-mouse F4/80, Alexa Fluor® 488 anti-mouse CD86 and APC anti-mouse CD206. Subsequently, the total cells were analyzed by FCM. F4/80^+^ cells were the total macrophages; and F4/80^+^CD86^+^ cells were M1-type macrophages; and F4/80^+^CD206^+^ cells were M2-type macrophages. The following formula (4) was used to calculate the total number of macrophages (*N*_total_) for per mg of tumor in the tumor tissues for different groups:

4 $$ M_{{{\text{total}}}} \left( {/{\text{mg of tumor}}} \right) \, = \, \left( {A_{{{\text{total}}}} \times P_{{{\text{F4}}/{8}0}} } \right) \, /T_{{{\text{weight}}}} $$

*A*_total_ represents the total number of cells in tumor tissues; *P*_F4/80_ represents the percentage of total macrophages (F4/80^+^ cells) in total cells in tumor tissues; *T*_weight_ represents the tumor weights.

After the in vivo antitumor efficacy study (on day 19 after the first administration), the blood serum of the mice was obtained, and the levels of immunogenic cytokines (TNF-α and IL-12) and immunosuppressed cytokines (IL-10 and TGF-β) in serum were measure by the ELISA kit. The levels of cytokines in blood serum were also measured at 48 h post the first administration by the ELISA kit.

The percentage of CD3^+^CD4^+^ T cells, CD3^+^CD8^+^ T cells and Treg in the tumors was evaluated by FCM. After the in vivo antitumor efficacy study (on day 19 after the first administration), tumor tissues were obtained, and then, tissues were ground, filtered by a copper network. After gradient centrifugated by Percoll, the cells were collected and counted, and stained with corresponding antibody markers for 1 h at 4 °C in dark. Then, the cells were analyzed by FCM. CD3^+^CD4^+^ T cells were marked with APC anti-mouse CD3 and FITC anti-mouse CD4; CD3^+^CD8^+^ T cells were marked with APC anti-mouse CD3 and PE anti-mouse CD8a, and Treg was marked with FITC anti-mouse CD4, PE anti-mouse CD25 and Alexa Fluor^®^ 647 anti-mouse FOXP3. CD3^+^ T cells represent the total T cells in tumor tissues. The following formula (5) was used to calculate the total number of T cells (*T*_total_) for per mg of tumor in the tumor tissues for different groups:

5 $$ T_{{{\text{total}}}} \left( {/{\text{mg of tumor}}} \right) \, = \, \left( {B_{{{\text{total}}}} \times P_{{{\text{CD3}}}} } \right) \, /T_{{{\text{weight}}}} $$

*B*_total_ represents the number of cells after centrifugation in tumor tissues; *P*_CD3_ represents the percentage of total T cells (CD3^+^ T cells) in tumor tissues; *T*_weight_ represents the tumor weights.

The macrophages phenotype, the percentage of CD3^+^ CD4^+^ T cells, CD3^+^ CD8^+^ T cells, and Treg in the tumors were evaluated on day 4 post the first administration, and the method was similar to the evaluation after the in vivo antitumor efficacy study.

### Dermal Sensitivity Test

The C57BL/6 mice and Kunming mice were injected intradermally with 0.1 mL saline, macrophages, and M1-type macrophages (3 × 10^6^ cells equivalent for the number of cells in antitumor efficacy study), respectively, and the method to obtain M1-type macrophages is shown in “2.7 Preparation of M/SLNP and M1/SLNP” part. The mice were observed and photographed at 24 h.

### Passive Cutaneous Anaphylaxis Test

Passive cutaneous anaphylaxis test was carried out. The C57BL/6 mice and Kunming mice were randomly divided into the negative control group, macrophages group and M1-type macrophages group and positive control group, respectively. Mice in each group were sensitized by intravenously injected with saline (negative control group), macrophages, and M1-type macrophages (3 × 10^6^ cells equivalent for the number of cells in antitumor efficacy study) and bovine serum albumin (5 mg, positive control group) every other day for four time. The method to obtain M1-type macrophages is shown in “2.7 Preparation of M/SLNP and M1/SLNP” part. The sensitized serum of the mice in each group was collected and was intradermally injected into the back of the mice, respectively. After 24 h, the mice injected with sensitized serum were intravenously injected with saline (negative control group), macrophages and M1-type macrophages (3 × 10^6^ cells equivalent for the number of cells in antitumor efficacy study) and bovine serum albumin (5 mg, positive control group), respectively. After 30 min, the skin on the back of the mice in each group was obtained and photographed.

### Statistical Analysis

The Student’s t-test was used to analyze the statistical comparisons between two groups, and differences were considered to be statistically significant when *p* < 0.05. All results were reported as the mean ± standard deviation (SD).

## Results and Discussion

### Characterization of M1/SLNP

**Fig. 1:**
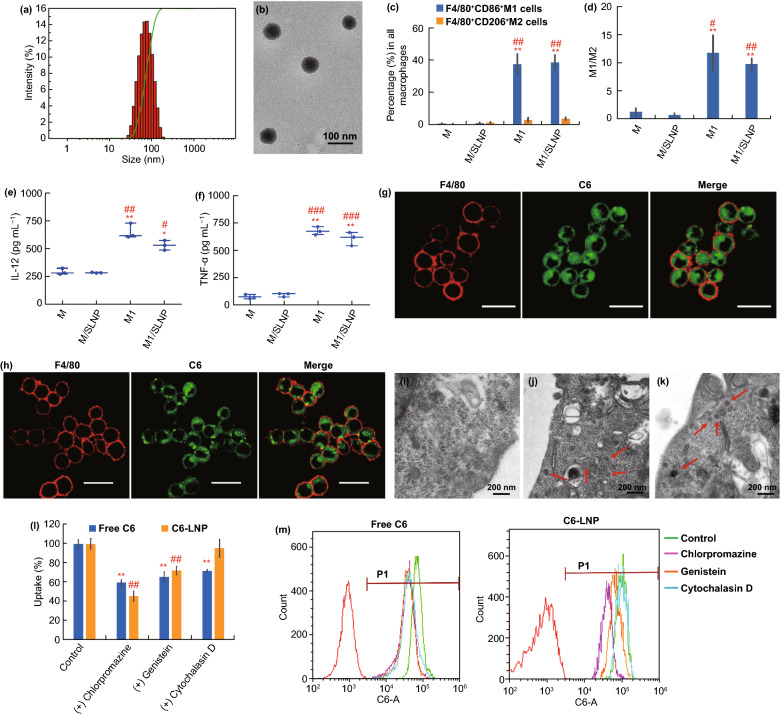
Characterization of M1/SLNP. **a** Particle size of SLNP. **b** TEM image of SLNP. **c** FCM analysis of phenotypes of M, M/SLNP, M1, and M1/SLNP in vitro*. *“Percentage (%)” in the Y-axis represents the percentage of M1-type macrophages (F4/80^+^ CD86^+^ M1 cells) in all macrophages or the percentage of M2-type macrophages (F4/80^+^ CD206^+^ M2 cells) in all macrophages. **d** Ratio of M1/M2 by FCM analysis. **p < 0.01, compared with M; #*p* < 0.05, ##*p* < 0.01, compared with M/SLNP. **e–f** Levels of **e** IL-12 and **f** TNF-α in vitro. ###*p* < 0.001, ##*p* < 0.01, #*p* < 0.05, compared with M/SLNP; **p < 0.01, *p < 0.05, compared with M. CLSM images of **g** M/C6-LNP and **h** M1/C6-LNP. Scale bar: 20 μm. TEM images of **i** macrophages, **j** M/SLNP**,** and **k** M1/SLNP. Red arrow indicated SLNP. Scale bar: 200 nm. The endocytic pathway of SF and SLNP in macrophages: **l** FCM histogram profiles of fluorescence intensity; **m** FCM analysis. *n* = 3, **p < 0.01, ##*p* < 0.01, compared with control

SLNPs were prepared by nanoprecipitation methods, and the optimal formulations of SLNP were determined by single factor assay on SF/soya lecithin mass ratio and soya lecithin concentration (Fig. S1). 12:100 and 7.5 mg mL^−1^ were determined as the optimal SF/soya lecithin mass ratio and soya lecithin concentration, respectively. SLNPs were successfully prepared with smaller particle size of 67.63 ± 5.02 nm, higher DL% of 5.58 ± 0.41% and PDI of 0.159 ± 0.018 (Fig. S1 and Table S1). The particle size and TEM image of SLNP are shown in Fig. 1a, b, respectively. As shown in Fig. 1b, SLNPs were nearly spherical particles and had good dispersibility.

M/SF, M/SLNP, and M1/SLNP were obtained by incubating macrophages with SF solution or SLNP. The optimal formulations of M/SF and M/SLNP were determined by single factor assays on SF concentration and incubation time (Fig. S2). 200 μg mL^−1^ and 2 h was determined as the optimal SF concentration and incubation time with higher drug loading and without cytotoxicity on macrophages, respectively (Fig. S2 and Table S2). The drug loading (μg/10 ^6^ cells) of M/SF, M/SLNP, and M1/SLNP was 24.46 ± 1.33, 37.43 ± 0.53, and 38.18 ± 0.80 μg/10^6^ cells, respectively (Table S2). The cellular uptake of SLNP was higher on macrophages compared with that of SF solution. The drug loading of M1/SLNP was similar to that of M/SLNP, indicating that M1-type macrophages did not affect the uptake of SLNP.

To better evaluate the macrophages phenotype and the impact of the loaded SLNP on macrophages phenotype, the proportions of M1-type macrophages and M2-type macrophages, and the ratio of M1-type macrophages to M2-type macrophages (M1/M2) were employed by FCM assay (Fig. 1c, d). The proportions of M1-type macrophages in M1-type macrophages (M1) group and M1/SLNP group were significantly higher than those in macrophages (M) group (*p* < 0.01, *p* < 0.01, respectively) and M/SLNP group (*p* < 0.01, *p* < 0.01, respectively) (Fig. 1c), and the ratios of M1/M2 in M1 group and M1/SLNP group were significantly higher than those in M group (*p* < 0.01, *p* < 0.01, respectively) and M/SLNP group (*p* < 0.05, *p* < 0.01, respectively) (Fig. 1d). The proportions of M1-type macrophages and the ratios of M1/M2 in M1 and M1/SLNP groups were comparable, suggesting that the loaded SLNP did not affect the macrophage phenotype. Specifically, we analyzed the levels of cytokines, including IL­12 and TNF­α, which were secreted by M1-type macrophages (Fig. 1e, f). The levels of IL­12 were increased in M1 group and M1/SLNP groups compared with that in M group (*p* < 0.01, *p* < 0.05, respectively) and M/SLNP group (*p* < 0.01, *p* < 0.05, respectively). The levels of TNF­ α were increased in M1 and M1/SLNP groups compared with that in M group (*p* < 0.01, *p* < 0.01, respectively) and M/SLNP group (*p* < 0.001, *p* < 0.001, respectively). The levels of cytokines were comparable in M1 group and M1/SLNP group, suggesting that the loaded SLNP did not affect the levels of cytokines secreted by M1-type macrophages. Collectively, these results indicated that macrophages were successfully polarized toward M1-type macrophages, and they could secrete cytokines including IL­12 and TNF­α, providing the theoretical basis for using M1-type macrophages as the therapeutic tool to exert immunotherapeutic antitumor efficacy.

M/C6-LNP and M1/C6-LNP were visualized under CLSM (Fig. 1g, h). LNPs were fluorescently labeled with the green fluorescence signal by loading C6. Macrophages membranes and M1-type macrophages membranes were labeled with red fluorescence signal using Alexa Fluor ® 647 anti-mouse F4/80 antibody. As shown in Fig. 1g, h, the green fluorescence signal of C6-LNP could be largely observed in macrophages or M1-type macrophages, indicating C6-LNPs were successfully loaded into macrophages or M1-type macrophages. In addition, macrophages, M/SLNP, and M1/SLNP were visualized under TEM (Fig. 1i–k). Spherical particles (red arrow) were observed in M/SLNP and M1/SLNP (Fig. 1j, k), while no similar spherical particles were observed in macrophages without loading SLNP (Fig. 1i), proving that the spherical particles represent SLNP loaded in M/SLNP or M1/SLNP. Collectively, these results indicate SLNPs were successfully loaded into macrophages or M1-type macrophages.

To investigate the endocytic pathway of free SF and SLNP in macrophages, macrophages were pre-incubated with chlorpromazine, genistein, and cytochalasin D, respectively, before loading them with either free C6 or C6-LNP (Fig. 1lm). The clathrin-dependent uptake was blocked by chlorpromazine, the caveolae-mediated endocytosis was inhibited by genistein, and macropinocytosis and phagocytosis were both inhibited by cytochalasin D. For free C6, cellular uptake in macrophages was inhibited by 40.36% (*p* < 0.01), 34.27% (*p* < 0.01), and 28.14% (*p* < 0.01) after treatment with chlorpromazine, genistein, and cytochalasin D, respectively, indicating that clathrin-mediated endocytosis, caveolae-mediated endocytosis, and macropinocytosis were all involved in the internalization process of free C6 in macrophages. For C6-LNP, cellular uptakes in macrophages were inhibited by 54.30% (*p* < 0.01) and 27.78% (*p* < 0.01) after treatment with chlorpromazine and genistein, respectively, indicating that the internalization process of C6-LNP in macrophages involved both clathrin-mediated endocytosis and caveolae-mediated endocytosis. Collectively, these results showed that the endocytic pathway of free C6 and C6-LNP was different in macrophages.

### SLNP Released from M1/SLNP and Exhibited Deep Tumor-penetrating Ability

The release profiles of SF from SF solution and SLNP are shown in Fig. S3a. SF was sustainably released from SF solution and SLNP in 72 h, respectively. The cumulative release of SF from SF solution at 72 h was 90.2% and 89.5% in pH 6.5 and pH 7.4, respectively. The cumulative release of SF from SLNP at 72 h was 55.8% and 56.8% in pH 6.5 and pH 7.4, respectively.

**Fig. 2:**
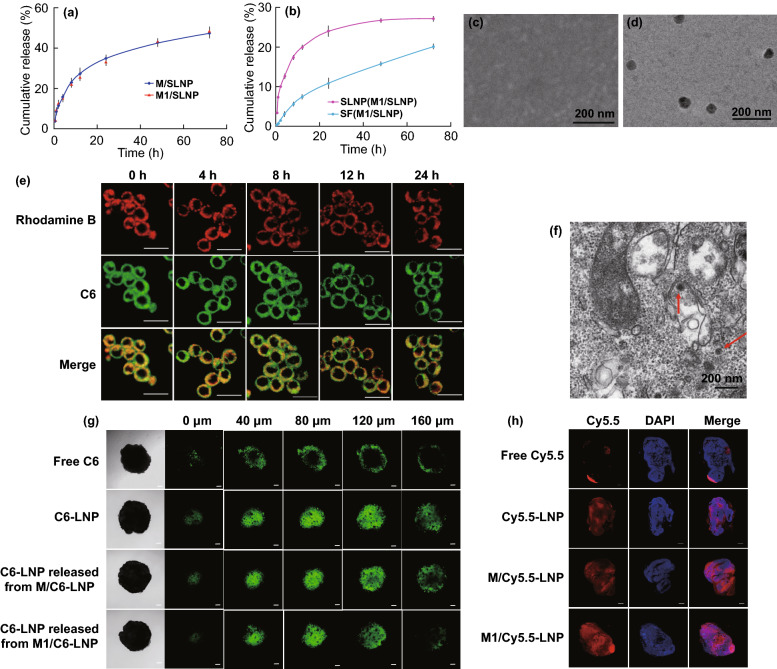
SLNP could be released from M1/SLNP and exhibited deep tumor-penetrating ability. **a** Release profiles of total SF from M/SLNP and M1/SLNP. **b** Release profiles of SF or SLNP from M1/SLNP, respectively. TEM images of released medium from **c** M1-type macrophages or **d** M1/SLNP. Scale bar: 200 nm. **e** CLSM images of M1/C6-LNP at 0, 4, 8, 12, and 24 h. Scale bar: 20 μm. **f** TEM images of M1/SLNP at 24 h. Scale bar: 200 nm. Red arrow indicated SLNP. **g** Penetration of M1/SLNP into tumor spheroids after incubation for 6 h. Scale bar: 100 μm. **h** Tumor sections of mice 24 h after injection of M1/Cy5.5-LNP, M/Cy5.5-LNP, Cy5.5-LNP and free Cy5.5, respectively, in vivo. Scale bar: 1000 μm

The release profiles of total SF from M/SLNP and M1/SLNP are shown in Fig. 2a. The cumulative release of total SF from M/SLNP and M1/SLNP at 72 h was 47.3% and 48.4%, respectively. The data indicated that SF could be released from M/SLNP and M1/SLNP; meanwhile, there were no significant differences between the cumulative release of total SF from macrophages and M1-type macrophages at 72 h and macrophages phenotype did not affect the release of SF. We further clarified whether SF would be released from M1/SLNP as the form of SF or SLNP using HPLC analysis (Fig. 2b) and TEM (Fig. 2d). SF and SLNP released from M1/SLNP were analyzed quantitatively using HPLC. The cumulative release of SF from M1/SLNP at 72 h was 20.19%, and the cumulative release of SLNP from M1/SLNP at 72 h was 27.17%. Among the total SF released from M1/SLNP, about 57.37% of the SF was released from M1/SLNP as SLNP and about 42.63% of the SF was released from M1/SLNP as SF (Fig. 2b). Spherical particles were observed in the released medium of M1/SLNP (Fig. 2d), while no similar spherical particles were observed in M1-type macrophages without loading SLNP (Fig. 2c), suggesting that the spherical particles represent SLNP released from M1/SLNP. In addition, HPLC analysis assay and TEM assay were also used to clarify whether SF would be released from M/SLNP as the form of SF or SLNP and the results were consistent with the foregoing analysis (Fig. S4a, b). These results indicated that both SF and SLNP would be released from M1/SLNP or M/SLNP.

The stability of C6-LNP in M1/C6-LNP was evaluated by fluorescence co-localization experiments (Fig. 2e). Lipids in LNP were labeled with rhodamine B (red). Green colors represent C6 loaded in LNP. M1/C6-LNPs were visualized under CLSM at 0, 4, 8, 12, and 24 h, respectively. Yellow orange fluorescence due to merging of red and green fluorescence was used to evaluate the co-localization efficiency. A high degree of co-localization from 0 to 24 h was observed in M1/C6-LNP, indicating that C6 was encapsulated in LNP, and C6-LNPs were stable when loaded in M1-type macrophages. In addition, M1/SLNPs at 24 h were visualized under TEM to further evaluate the stability of SLNP (Fig. 2f). As shown in Fig. 2f, the spherical particles indicated by a red arrow were observed and represent SLNP loaded in M1/SLNP. The stability of SLNP in M/SLNP was also evaluated (Fig. S5a, b). These results indicate that SLNP could were stably loaded as spherical particles in the macrophages and M1-type macrophages.

We explored the deep tumor-penetrating ability of SLNP, SLNP released from M/SLNP, and SLNP released from M1/SLNP, respectively, in vitro by constructing a three-dimensional (3D) multicellular tumor spheroid model (Fig. 2g). LNPs were labeled by loading C6 (green). Free C6, C6-LNP, C6-LNP released from M/C6-LNP and C6-LNP released from M1/C6-LNP were incubated with tumor spheroids for 6 h, respectively. The green fluorescence signal in C6-LNP, M/C6-LNP, and M1/C6-LNP group was visualized to penetrate gradually into the tumor at a depth of 120 µm, respectively, and uniformly distributed in most areas of the tumor. Comparatively, at such a depth, the green fluorescence signal in the free C6 group was only observed on the periphery of the tumor spheroid. Collectively, the results suggested that C6-LNP and the C6-LNP released from M/C6-LNP and M1/C6-LNP exhibited deep tumor-penetrating ability. Next, the tumor-penetrating ability of SLNP, M/SLNP, and M1/SLNP was also explored in vivo. Tumor sections were obtained after injection of M1/Cy5.5-LNP, M/Cy5.5-LNP, Cy5.5-LNP or free Cy5.5 at 24 h, respectively (Fig. 2h). As shown in Fig. 2h, the red fluorescence signal in the Cy5.5-LNP group, M/Cy5.5-LNP group, and M1/Cy5.5-LNP group was visualized penetrating into the middle of tumor. The red fluorescence signal in free Cy5.5 group was seen only on the periphery of the tumor sections. Above results suggested that Cy5.5-LNP, M/Cy5.5-LNP, and M1/Cy5.5-LNP exhibited deep tumor-penetrating ability in vivo.

The in vitro cellular uptake studies for free C6, C6-LNP, C6-LNP released from M/C6-LNP, and C6-LNP released from M1/C6-LNP were studied on Hepa1-6 cells by fluorescence microscopy (Fig. S6a) and FCM analysis (Fig. S6b, c) after 0.5- and 2-h incubation. As shown in Fig. S6a, green fluorescence boosted up with increasing incubation time, indicating that free C6, C6-LNP, C6-LNP released from M/C6-LNP and C6-LNP released from M1/C6-LNP could be internalized into Hepa1-6 cells efficiently. The cellular mean fluorescence intensity (MFI) calculated from FCM data showed similar results in Fig. S6b, c.

### M1/SLNP Enhanced the Tumor Targeting Delivery

The migration ability of M, M1, M/SLNP, and M1/SLNP toward Hepa1-6 cells in vitro is displayed in Fig. S7a. As shown in Fig. S7a, few macrophages were observed at the lower chamber of the Transwell when DMEM media were added to the lower chamber. Cells migrated across the Transwell membrane to the lower chamber of the Transwell which significantly increased when conditioned media of Hepa1-6 cells were added to the lower chamber. These results proved the tumor targeting ability of macrophages and M1-type macrophages, and provided the theoretical basis for using macrophages and M1-type macrophages as the tumor targeting vessel.

**Fig. 3:**
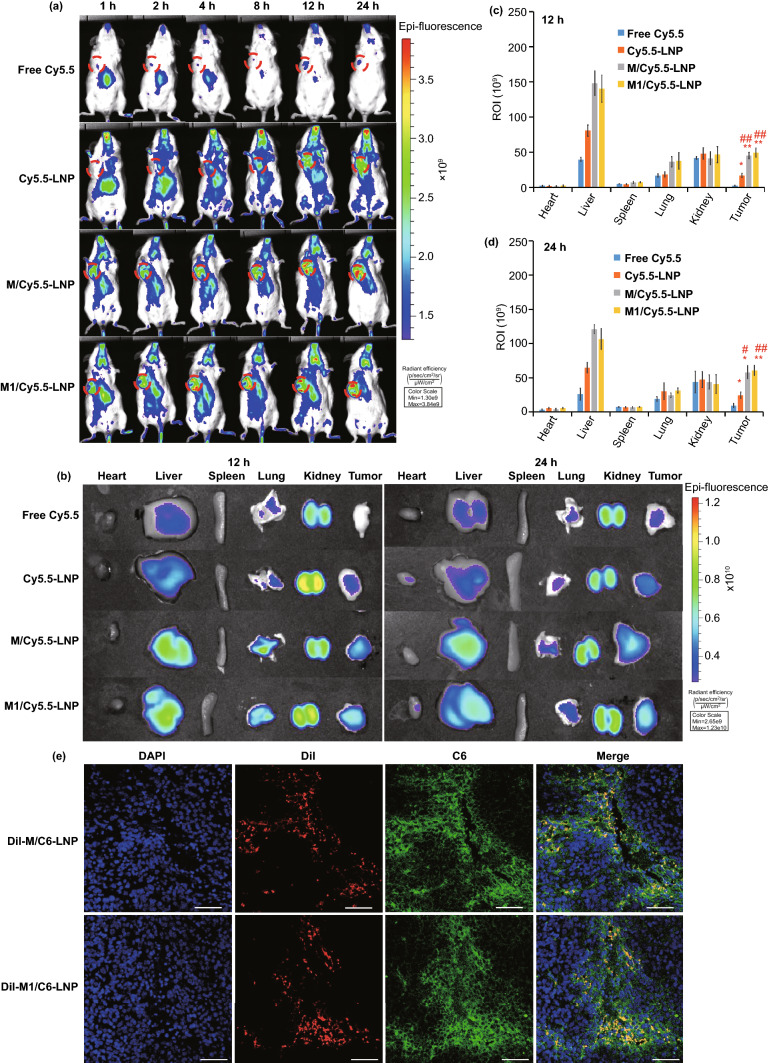
M1/SLNP enhanced the tumor targeting delivery. **a** In vivo imaging at 1, 2, 4, 8, 12, and 24 h post-intravenous injection of M1/Cy5.5-LNP, M/Cy5.5-LNP, Cy5.5-LNP, and free Cy5.5, and tumors were marked with red circles. **b** Ex vivo imaging after the mice were dissected at 12 h and at 24 h post-administration. **c-d** Radiant efficiency at 12 h and at 24 h based on the ex vivo results. ##*p* < 0.01, #*p* < 0.05, compared with Cy5.5-LNP group; **p < 0.01, *p < 0.05, compared with free Cy5.5 group. **e** CLSM images of the tumor tissue section after intravenous injection of DiI-M/C6-LNP and DiI-M1/C6-LNP at 24 h. Scale bar: 50 μm

The real-time biodistribution and tumor targeting ability of M1/Cy5.5-LNP, M/Cy5.5-LNP, Cy5.5-LNP, and free Cy5.5 were evaluated in Hepa1-6 tumor-bearing mice, respectively. As shown in Fig. 3a, the fluorescence signal in tumor tissues in Cy5.5-LNP group, M/Cy5.5-LNP group, and M1/Cy5.5-LNP group was higher than that in free Cy5.5 group and the fluorescence signal in tumor tissues in M/Cy5.5-LNP group and M1/Cy5.5-LNP group was higher than that in Cy5.5-LNP group, suggesting that M/Cy5.5-LNP and M1/Cy5.5-LNP enhanced the tumor targeting delivery. In addition, the fluorescence signal in the tumor tissues was observed in M/Cy5.5-LNP group and M1/ Cy5.5-LNP group after intravenous administration for 1 h and the fluorescence signal in free Cy5.5 group and Cy5.5-LNP group was nearly invisible in the tumor tissues, indicating that macrophages and M1-type macrophages could reach tumor tissues earlier than the free Cy5.5 and Cy5.5-LNP. Ex vivo imaging assay was performed at 12 h (Fig. 3b, c and Table. S3) and 24 h post-administration (Fig. 3b, d and Table S4). As shown in Fig. 3c, the fluorescence signal intensity of M/Cy5.5-LNP group and M1/ Cy5.5-LNP group in the tumor tissues at 12 h was significantly enhanced than that in the free Cy5.5 group (*p* < 0.01, *p* < 0.01, respectively) and Cy5.5-LNP group (*p* < 0.01, *p* < 0.01, respectively). As shown in Fig. 3d, the fluorescence signal intensity of M/Cy5.5-LNP group and M1/Cy5.5-LNP group in the tumor tissues at 24 h was significantly enhanced compared with free Cy5.5 group (*p* < 0.05, *p* < 0.01, respectively) and Cy5.5-LNP group (*p* < 0.05, *p* < 0.01, respectively). These data indicated that M/Cy5.5-LNP and M1/Cy5.5-LNP could enhance the tumor targeting delivery. As shown in Table S3, the tumor targeting efficiency of M/Cy5.5-LNP group and M1/ Cy5.5-LNP group at 12 h was significantly enhanced compared with free Cy5.5 group (*p* < 0.01, *p* < 0.01, respectively) and Cy5.5-LNP group (*p* < 0.05, *p* < 0.05, respectively). As shown in Table S4, the tumor targeting efficiency of M/Cy5.5-LNP group and M1/ Cy5.5-LNP group at 24 h was significantly enhanced compared with free Cy5.5 group (*p* < 0.01, *p* < 0.05, respectively) and Cy5.5-LNP group (*p* < 0.01, *p* < 0.05, respectively). The tumor targeting efficiency between M/Cy5.5-LNP group and M1/ Cy5.5-LNP group in the tumor tissues did not have significant deference. These results indicated that M/Cy5.5-LNP and M1/Cy5.5-LNP could target more selectively the tumor compared with free Cy5.5 group and Cy5.5-LNP group, and the tumor targeting ability of M/Cy5.5-LNP and M1/Cy5.5-LNP was comparable.

The tumor targeting ability of M/SLNP and M1/SLNP in vivo was further determined using CLSM images of the tumor tissue section after intravenous administration of DiI-M/C6-SLNP or DiI-M1/C6-SLNP for 24 h, respectively (Fig. 3e). Macrophages and M1-type macrophages were labeled with DiI (red). LNPs were labeled by loading C6 (green). The nuclei were stained with DAPI (blue). The red fluorescence signal was observed in the tumor tissues, indicating that macrophages and M1-type macrophages could actively target the tumor tissues. The green fluorescence signal was observed in the sites of tumor, suggesting that C6-LNPs were successfully delivered to tumor tissues by macrophages and M1-type macrophages. In addition, green fluorescence signal was observed in other cells besides administrated macrophages and M1-type macrophages, indicating that C6-LNP could be released from M/C6-LNP and M1/C6-LNP in the tumor tissues in vivo. These results proved that M/C6-LNP and M1/C6-LNP exhibited tumor targeting ability in vivo and the loaded drug could be released from macrophages and M1-type macrophages in the tumor tissues in vivo, providing the theoretical basis for using M1-type macrophages as the tumor targeting biomimetic vessel.

### M1/SLNP Enhanced Antitumor Efficacy in vitro and in vivo

**Fig. 4:**
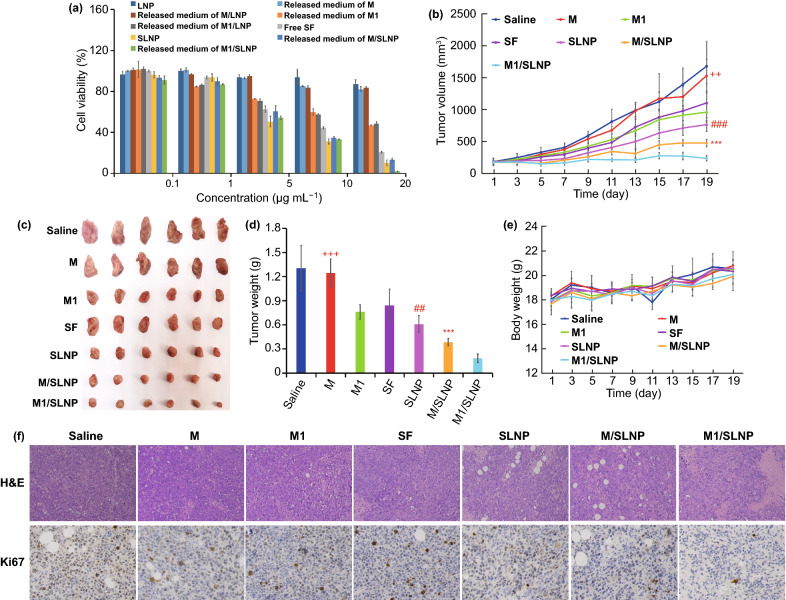
M1/SLNP enhanced antitumor efficacy in vitro and in vivo. **a** Cell viability of M1/SLNP in Hepa1-6 cells in vitro*.* **b** In vivo tumor volume changes. **c** Photographs of tumors. **d** Tumor weights. **e** Body weight changes from Hepa1-6-bearing mice treated with NS, M, M1, free SF, SLNP, M/SLNP, and M1/SLNP via the tail vein. **f** H&E and Ki67 results of tumor tissues. Magnification: H&E 200× , Ki67 200× . +++*p* < 0.001, ++*p* < 0.01, compared with M1; ##*p* < 0.01, ###*p* < 0.001, compared with M/SLNP; ***p < 0.001, compared with M1/SLNP, n = 6

The antitumor efficacy was investigated by MTT assay in vitro (Fig. 4a). LNP, released medium of macrophages (released medium of M), and released medium of M/LNP (released medium of M/LNP) exhibited over 80% cell viability. Released medium of M1-type macrophages (released medium of M1) and released medium of M1/LNP (released medium of M1/LNP) showed cytotoxicity, which were caused by M1-type macrophages. These results indicated that M1-type macrophages could display immunotherapeutic antitumor efficacy as therapeutic tool. Both free SF, SLNP, released medium of M/SLNP, and released medium of M1/SLNP exhibited cytotoxicity. The half maximal inhibitory concentration (IC_50_) of free SF, SLNP, released medium of M1, released medium of M1/LNP, released medium of M/SLNP, and released medium of M1/SLNP was 7.62 ± 0.37, 4.62 ± 0.55, 11.74 ± 0.12, 12.33 ± 0.89, 5.13 ± 0.52, and 2.40 ± 0.23 μg mL^−1^, respectively (Table S5). The IC_50_ of released medium of M1/SLNP was significantly lower compared with SLNP (*p* < 0.05) and released medium of M/SLNP (*p* < 0.01), suggesting that M1-type macrophages provided an advantage in improving the cytotoxicity of SLNP and enhancing antitumor efficacy in vitro.

The therapeutic efficiency of M1/SLNP was evaluated in vivo. As shown in Fig. 4b, the tumor volumes in the M/SLNP group were smaller than those in the SF solution group (*p* < 0.001), SLNP group (*p* < 0.001), which could be contributed to the high tumor targeting efficacy of macrophages. M1 significantly inhibited tumor growth compared with M group (*p* < 0.01), and M1/SLNP showed better antitumor efficacy compared with M/SLNP (*p* < 0.001), due to the immunotherapeutic antitumor efficacy of M1-type macrophages as therapeutic tool. M1/SLNP significantly inhibited the tumor growth compared with SLNP group (*p* < 0.01), which exhibited the best antitumor efficiency, indicating that M1/SLNP improved the antitumor efficacy of SLNP. These results could be attributed to both the high tumor targeting efficacy and the immunotherapeutic antitumor efficacy of M1-type macrophages. The tumor growth inhibition rates of M, M1, free SF, SLNP, M/SLNP, and M1/SLNP group were 8.84%, 42.49%, 33.89%, 54.04%, 71.09%, and 85.02%, respectively. Excised tumors were photographed (Fig. 4c) and weighed (Fig. 4d), and the results were in accordance with the tumor volume results. As shown in Fig. 4f, M1/SLNP exhibited the lowest tumor-cell proliferation rate and the highest tumor necrosis level. The variation of the relative body weights of the mice is shown in Fig. 4e. Body weights in M1/SLNP group showed no serious reduction during treatment period (*p* > 0.05), suggesting the low systemic toxicity of M1/SLNP. The preliminary safety of the carrier was investigated by immunohistochemistry evaluation, dermal sensitivity test, and passive cutaneous anaphylaxis test. Pathological changes or inflammatory infiltrates were not observed in organ tissues (Fig. S15a), indicating that M1/SLNP exhibited biocompatibility without toxicities to normal tissue. The dermal sensitivity test showed that the intradermal injection area of the mice in macrophages group and M1-type macrophages group did not show any obvious erythema and swelling (Figs. S16, S17). The passive cutaneous anaphylaxis test indicated that macrophages group and M1-type macrophages group showed no allergic reaction, as no blue blot was detected (Figs. S18, S19).

### M1/SLNP Relieved the Immunosuppressive Tumor Microenvironments

**Fig. 5:**
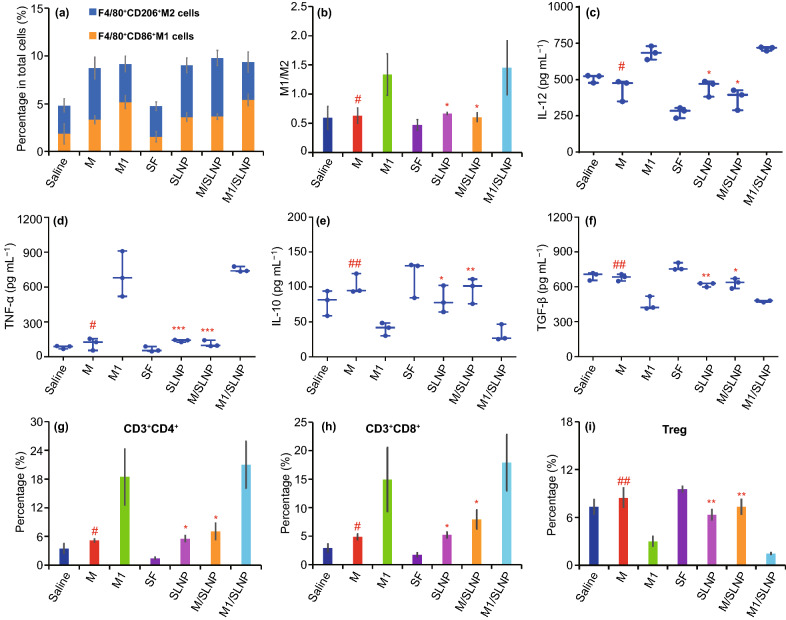
M1/SLNP relieved the immunosuppressive tumor microenvironments. The analysis of macrophages, CD3^+^CD4^+^ T cells, CD3^+^CD8^+^ T cells and Treg in tumor tissues after the in vivo antitumor efficacy study. Quantitative analysis of M1-type macrophages and M2-type macrophages in tumor tissues by FCM: **a** percentage of M1-type macrophages (F4/80^+^CD86^+^ M1 cells, blue bar chart) in total cells in tumor tissues and the percentage of M2-type macrophages (F4/80^+^CD206^+^ M2 cells, yellow bar chart) in total cells in tumor tissues after treatment with formulations (NS, M, M1, free SF, SLNP, M/SLNP, and M1/SLNP); **b** ratio of M1/M2. The levels of cytokines in blood serum: **c** IL-12; **d** TNF-α; **e** IL-10; **f** TGF-β. **g-i** Percentage of CD3^+^CD4^+^ T cells **(g)**, CD3^+^CD8^+^ T cells **(h),** and Treg **(i)**. ##*p* < 0.01, #*p* < 0.05, compared with M1; ***p < 0.001, **p < 0.01, *p < 0.05, compared with M1/SLNP, n = 3

Macrophages in the tumor tissues were analyzed by FCM assay after treatment with different formulations (Fig. 5a, b). Compared with M group, the percentage of M1-type macrophages in total cells in tumor tissues was higher in M1 group (Fig. 5a). The percentage of M1-type macrophages in total cells in tumor tissues was higher in M1/SLNP group compared with M/SLNP group (Fig. 5a). Higher ratio of M1/M2 in M1 group was detected compared with M group (*p* < 0.05), and the ratio of M1/M2 in M1/SLNP group was higher compared with M/SLNP group (*p* < 0.05) (Fig. 5b). These results suggested that M1 and M1/SLNP increased the percentage of M1-type macrophages in total cells in tumor tissues and further changed the ratio of M1/M2 in the tumor microenvironments through the immunomodulation of M1-type macrophages. The total number of macrophages for per mg of tumor in the tumor tissues for different groups after the in vivo antitumor efficacy study is shown in Fig. S8. The macrophages phenotype was evaluated on day 4 post the first administration (Fig. S10a, b). The results showed that M1/SLNP could increase the percentage of M1-type macrophages in total cells in tumor tissues and changed the ratio of M1/M2 in the tumor microenvironments through the immunomodulation of M1-type macrophages within the first 4 days post the first administration.

The cytokines were measured after treatment with different formulations (Fig. 5c–f). Higher levels of IL-12 and TNF-α in M1 group were detected compared with M group (*p* < 0.05, *p* < 0.05, respectively). The levels of IL-12 and TNF-α in M1/SLNP group were higher than those in SLNP group (*p* < 0.05, *p* < 0.001, respectively). The levels of IL-12 and TNF-α in M1/SLNP group were higher than those in M/SLNP group (*p* < 0.05, *p* < 0.001, respectively). Lower levels of IL-10 and TGF-β in M1 group were detected compared with M group (*p* < 0.01, *p* < 0.01, respectively). Lower levels of IL-10 and TGF-β in M1/SLNP group were detected compared with SLNP group (*p* < 0.05, *p* < 0.01, respectively). Lower levels of IL-10 and TGF-β in M1/SLNP group were detected compared with M/SLNP group (*p* < 0.01, *p* < 0.05, respectively). These findings indicated that the immunogenic cytokines increased and immunosuppressed cytokines decreased after the administration of M1-type macrophages. The levels of cytokines (IL-12, TNF-α, IL-10, and TGF-β) in blood serum were also measured at 48 h post the first administration (Fig. S11), and the results indicated that IL-12 and TNF-α increased after the administration of M1/SLNP within the first 48 h post the first administration. The levels of IL-10 and TGF-β in different groups did not have significant difference.

The percentage of CD3^+^CD4^+^ T cells, CD3^+^CD8^+^ T cells, and Treg in the tumors after treatment with different formulations was measured after gradient centrifugated by Percoll, respectively (Fig. 5g–i and S12–S14). The percentage of CD3^+^CD4^+^ T cells and CD3^+^CD8^+^ T cells in M1 group was higher than that in M group (*p* < 0.05, *p* < 0.05, respectively). Compared with M group, the percentage of Treg in M1 group was lower (*p* < 0.01). The percentage of CD3^+^CD4^+^ T cells and CD3^+^CD8^+^ T cells in the M1/SLNP group was higher than that in SLNP group (*p* < 0.05, *p* < 0.05, respectively). Compared with SLNP group, the percentage of Treg in M1/SLNP group was lower (*p* < 0.01). These results indicated that the percentage of CD3^+^CD4^+^ T cells and CD3^+^CD8^+^ T cells was increased and the percentage of Treg was reduced in M1 group and M1/SLNP group through the immunomodulation of M1-type macrophages. The total number of T cells for per mg of tumor in the tumor tissues for different groups after the in vivo antitumor efficacy study is shown in Fig. S9. Besides, the percentage of CD3^+^CD4^+^ T cells, CD3^+^CD8^+^ T cells, and Treg in the tumor tissues was analyzed on day 4 post the first administration (Fig. S10c–e). The results showed that the percentage of CD3^+^CD4^+^ T cells, CD3^+^CD8^+^ T cells and Treg in different groups did not have significant difference.

Collectively, the evaluation of macrophages, CD3^+^CD4^+^ T cells, CD3^+^CD8^+^ T cells, Treg and cytokines after treatment with different formulations suggested that M1/SLNP could relieve the immunosuppressive tumor microenvironments and M1-type macrophages could be used as the therapeutic tool to display immunotherapeutic antitumor efficacy and improve the chemotherapy antitumor efficacy.

## Conclusion

In summary, we developed a M1-type macrophage-based treatment and drug delivery system which promoted the tumor targeting delivery and antitumor efficacy. M1-type macrophages as therapeutic tool displayed immunotherapeutic antitumor efficacy. Meanwhile, M1-type macrophages as drug delivery vessel exhibited tumor targeting ability. Importantly, we demonstrated that M1-type macrophages could significantly increase the accumulation of SF in tumor sites and enhance tumor targeting delivery (*p* < 0.01). M1/SLNP showed a superior antitumor effect with obvious tumor suppression. Overall, M1-type macrophages-based treatment and drug delivery system might provide a new potential strategy for the development of cell therapy.

## Electronic Supplementary Material

Below is the link to the electronic supplementary material.

Supplementary material 1 (PDF 1486 kb)
