# Supplementary material for: Nanoparticle-Loaded Polarized-Macrophages for Enhanced Tumor Targeting and Cell-Chemotherapy
Source: Nanomicro Lett. 2020 Oct 27;13:6. doi: 10.1007/s40820-020-00531-0 (PMC8187668; doi:10.1007/s40820-020-00531-0)
Supplement: Supplementary file 1 — Supplementary material 1 (PDF 1486 kb) [file 40820_2020_531_MOESM1_ESM.pdf]

Supporting Information for

Nanoparticle-Loaded Polarized-Macrophages for Enhanced Tumor Targeting and Cell-Chemotherapy

Teng Hou[1], Tianqi Wang[1], Weiwei Mu[1], Rui Yang[1], Shuang Liang[1], Zipeng Zhang[1], Shunli Fu[1], Tong Gao[1], Yongjun Liu[1, *], Na Zhang[1, *]

[1]Department of Pharmaceutics, Key Laboratory of Chemical Biology (Ministry of Education), School of Pharmaceutical Sciences, Cheeloo College of Medicine, Shandong University, 44 Wenhuaxi Road, Jinan 250012, People's Republic of China

*Corresponding authors. E-mail: liuyongjun@sdu.edu.cn (Yongjun Liu); zhangnancy9@sdu.edu.cn (Na Zhang)

S1 Supplementary Figures

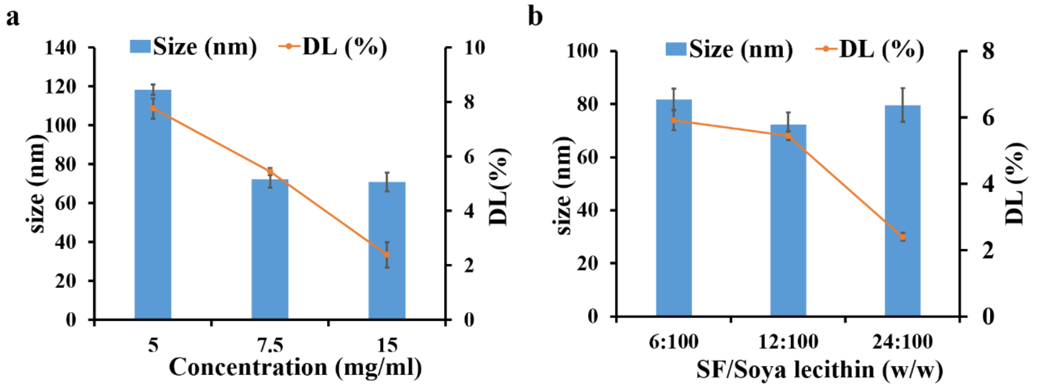

Fig. S1 Single factor study of SLNP. **a-b** Single factor study and characterization of SLNP. **a** Particle sizes and drug-loading efficiency of SLNP at various concentration of soya lecithin; **b** Particle sizes and drug-loading efficiency of SLNP at various ratio of SF/ soya lecithin (w/w). Data were given as mean ± SD (n = 3)

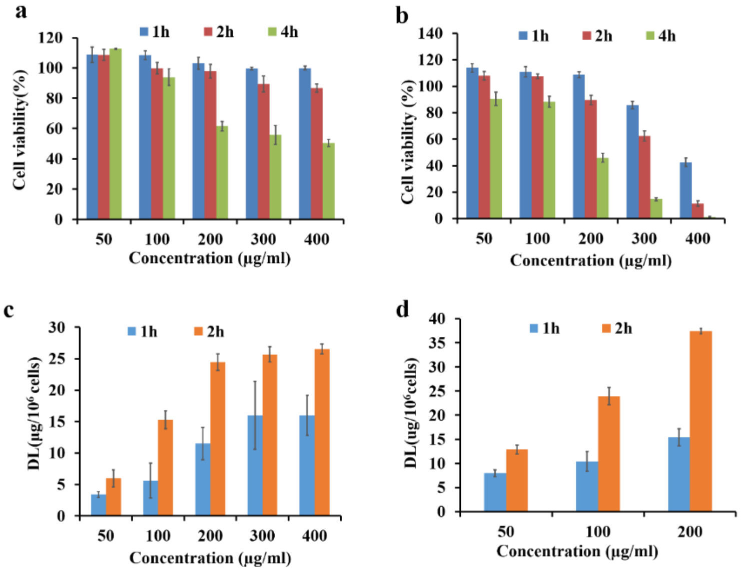

Fig. S2 Single factor study of M/SF and M/SLNP. **a** Cell viability of SF solution in macrophages at various concentration of SF and incubation time. **b** Cell viability of SLNP in macrophages at various concentration of SF and incubation time. **c** Drug-loading of macrophages for SF solution at various concentration of SF and incubation time. **d** Drug-loading of macrophages for SLNP at various concentration of SF and incubation time

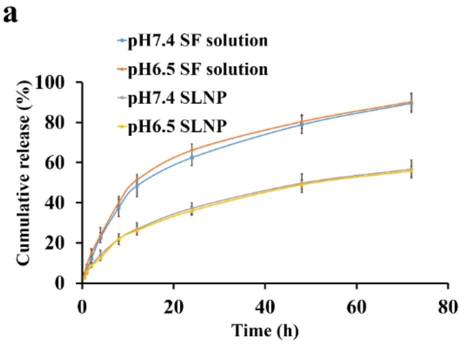

Fig. S3 **a** Release profiles of SF from SF solution and SLNP (n = 3)

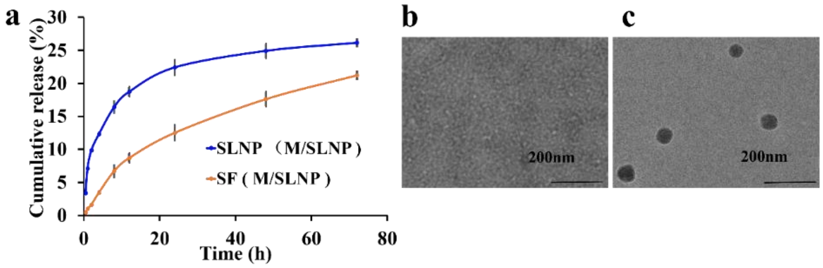

Fig. S4 **a** Release profiles of SF or SLNP from M/SLNP, respectively. **b-c** TEM images of released medium from macrophages **(b)** or M/SLNP **(c)**. Scale bar: 200 nm

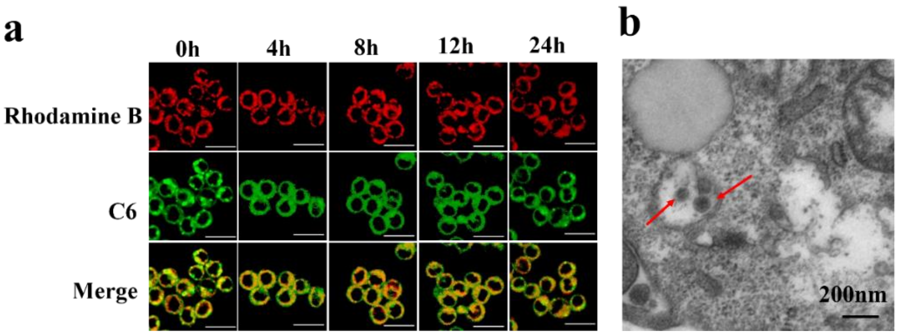

Fig. S5 a CLSM images of M/C6-LNP at 0, 4, 8, 12 and 24 h. Scale bar: 20 μm. LNP were labeled by Rhodamine B (red). Green colors represent C6 loaded in LNP. **b** TEM images of M/SLNP at 24 h. Scale bar: 200 nm. Red arrow indicated SLNP

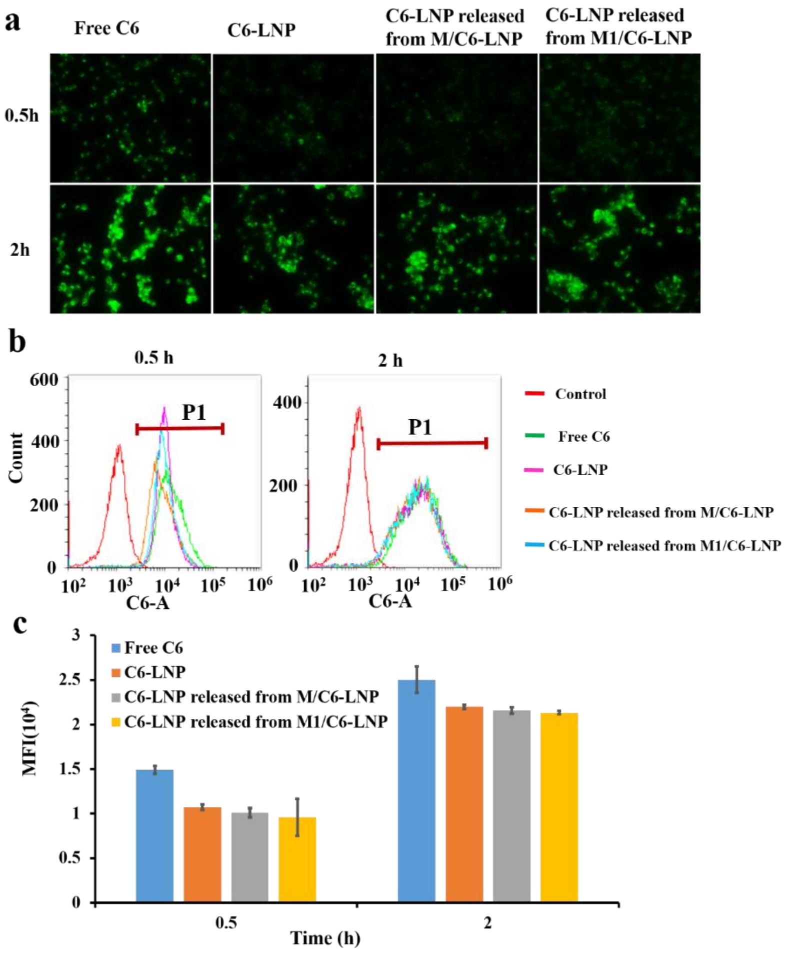

Fig. S6 Cellular uptake study of Hepa1-6 cells incubated with free C6, C6-LNP, C6-LNP released from M/C6-LNP and C6-LNP released from M1/C6-LNP for 0.5 and 2 h. **a** Inverted fluorescence microscope images. Magnification: 20×. **b** FCM analysis. **c** Quantitative analysis of cellular level of mean fluorescence intensity by FCM, n = 3

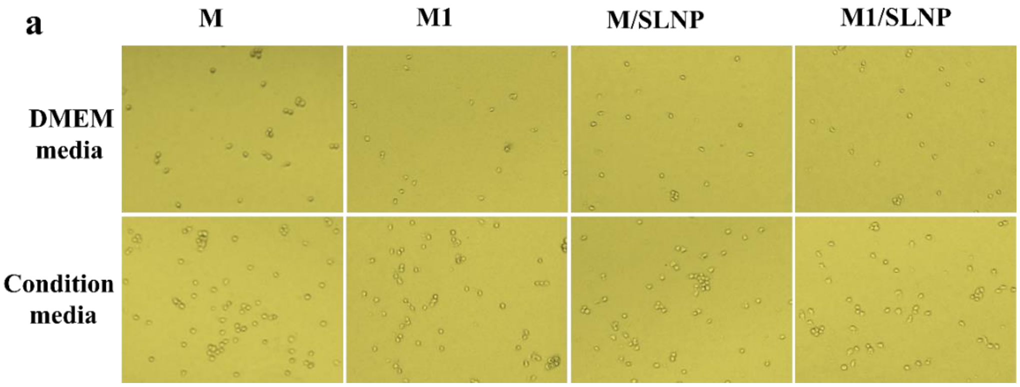

Fig. S7 Tumor-targeting activity of M, M1, M/SLNP and M1/SLNP towards Hepa1-6 cells *in vitro*. Images of macrophages or M1-type macrophages transported in the lower chamber of the transwell system in the presence of DMEM media or conditioned media of Hepa1-6 cells. Magnification: 20×

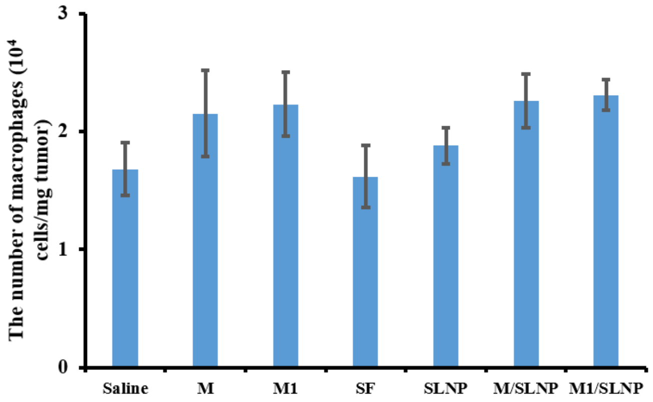

Fig. S8 Total number of macrophages for per mg of tumor in the tumor tissues for different groups after the *in vivo* antitumor efficacy study

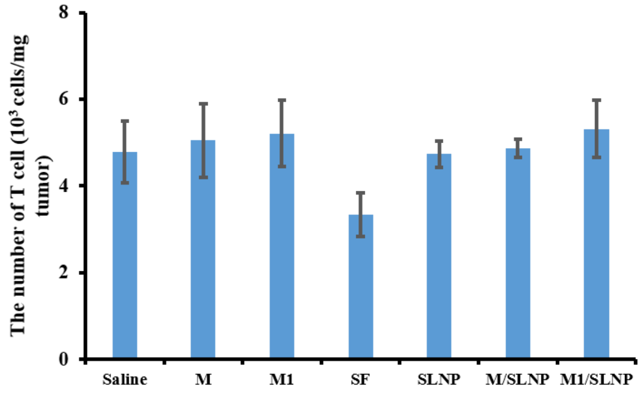

Fig. S9 Total number of T cells for per mg of tumor in the tumor tissues for different groups after the *in vivo* antitumor efficacy study

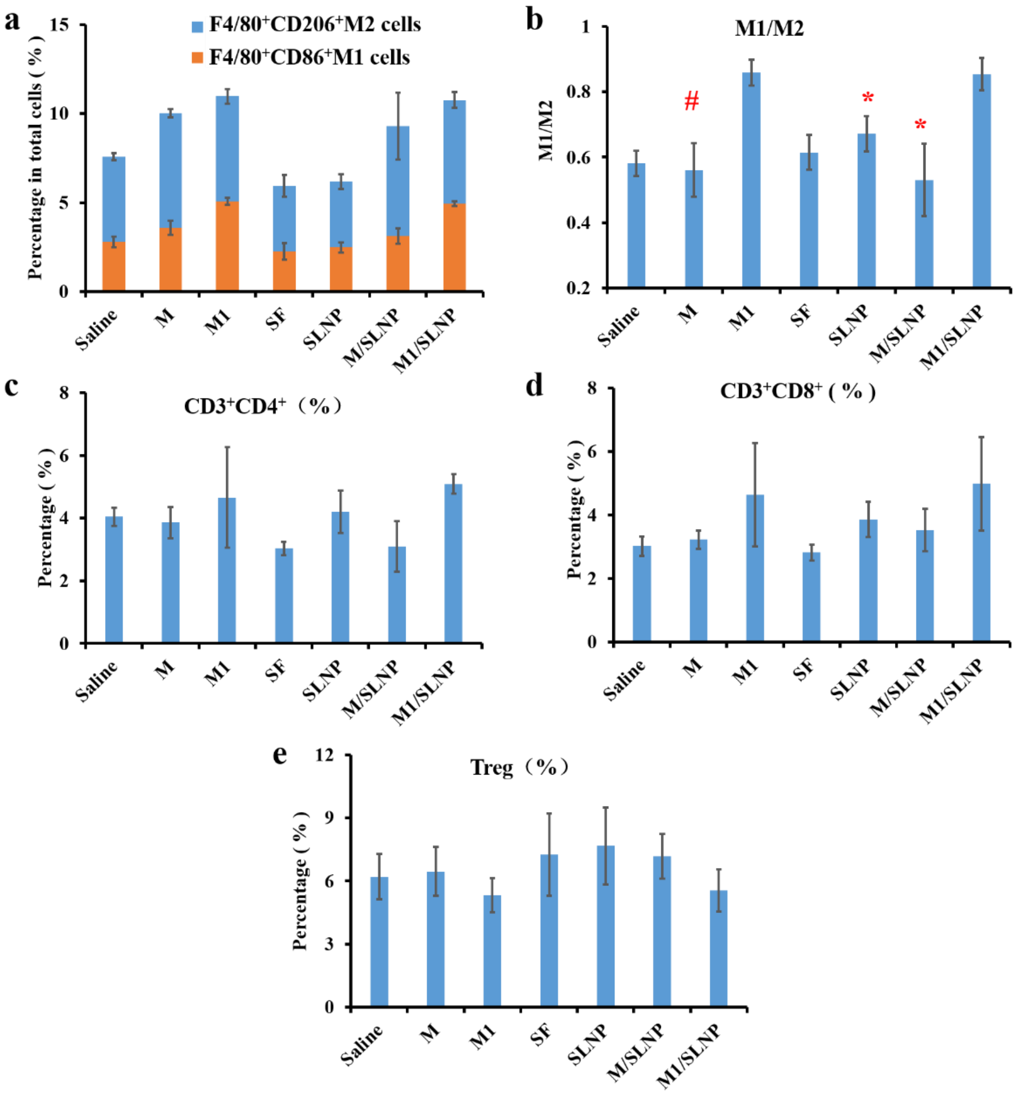

Fig. S10 Analysis of macrophages, $CD3^+CD4^+$ T cells, $CD3^+CD8^+$ T cells and Treg in tumor tissues on day 4 post the first administration. **a-b** Quantitative analysis of M1-type macrophages and M2-type macrophages in tumor tissues by FCM. **a** Percentage of M1-type macrophages ($F4/80^+CD86^+$ M1 cells, blue bar chart) in total cells in tumor tissues and the percentage of M2-type macrophages ($F4/80^+CD206^+$ M2 cells, yellow bar chart) in total cells in tumor tissues after treatment with formulations (NS, M, M1, free SF, SLNP, M/SLNP and M1/SLNP); **b** Ratio of M1/M2. **c-e** Proportion of $CD3^+CD4^+$ T cells **(c)**, $CD3^+CD8^+$ T cells **(d)** and Treg **(e)**. #$p < 0.05$, compared with M1; *$p < 0.05$, compared with M1/SLNP, n = 3

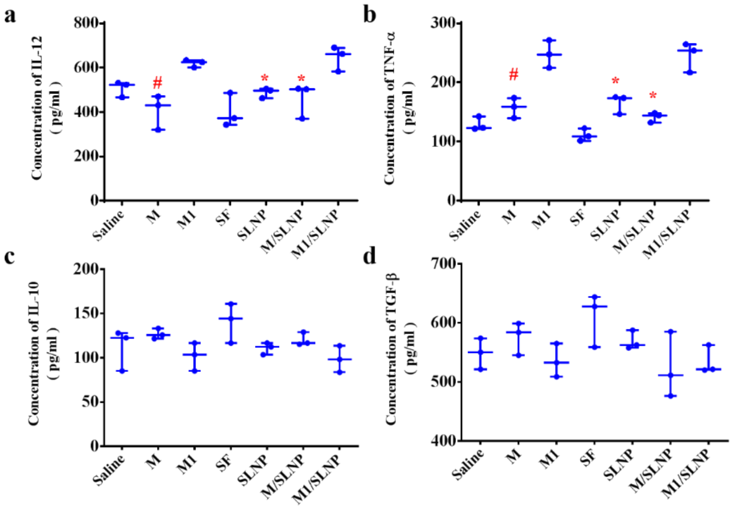

Fig. S11 Levels of cytokines in blood serum at 48 h post the first administration. (**a**) IL-12; (**b**) TNF-α; (**c**) IL-10; (**d**) TGF-β. #$p < 0.05$, compared with M1; *$p < 0.05$, compared with M1/SLNP, n = 3

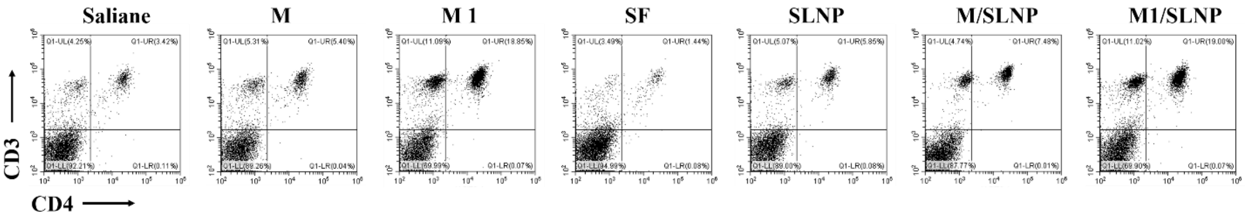

Fig. S12 FCM results of the percentage of $CD3^{+}CD4^{+}$ T cells in tumor tissues for different groups

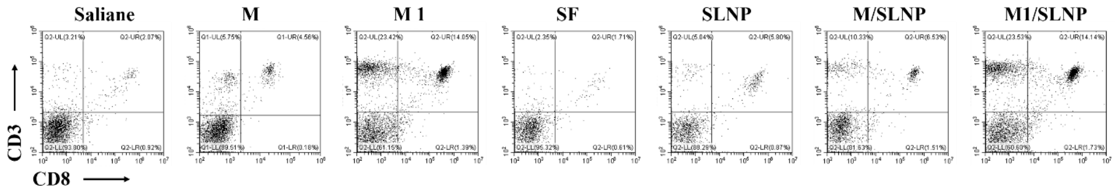

Fig. S13 FCM results of the percentage of $CD3^{+}CD8^{+}$ T cells in tumor tissues for different groups

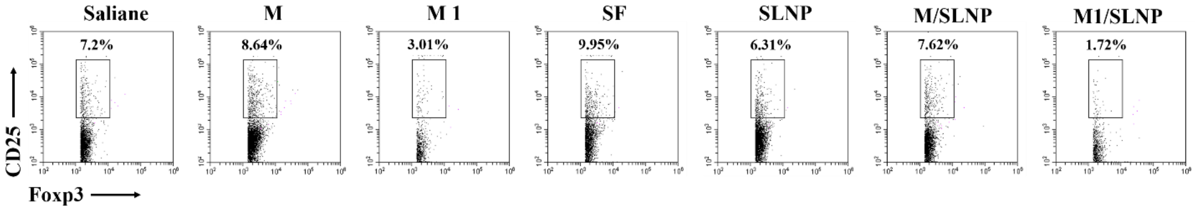

Fig. S14 FCM results of the percentage of Treg in tumor tissues for different groups

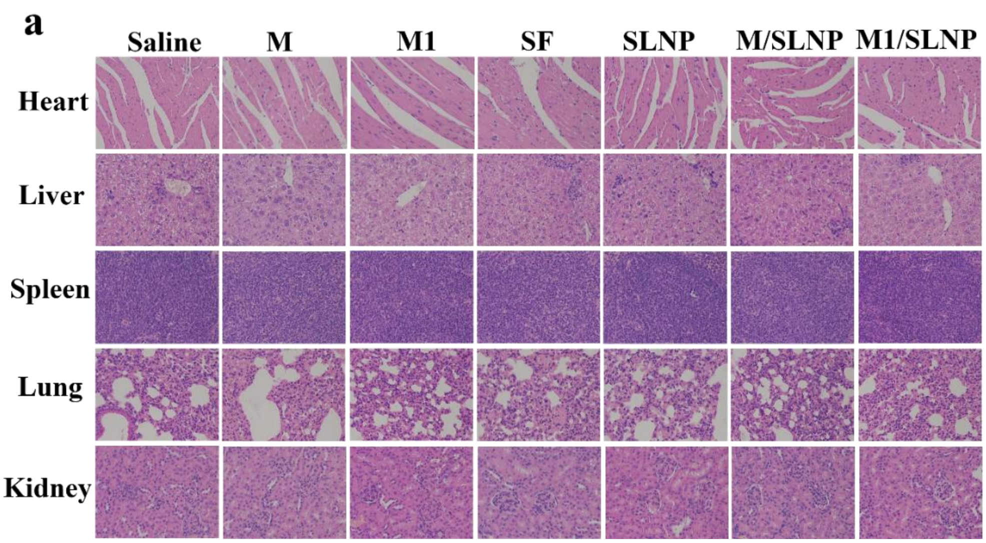

Fig. S15 Immunohistochemical analysis. Representative microscopy images of H&E-stained histological sections after treatment with saline, M, M1, SF, SLNP, M/SLNP and M1/SLNP, respectively. Magnification: heart, liver, spleen, lung and kidney 200×

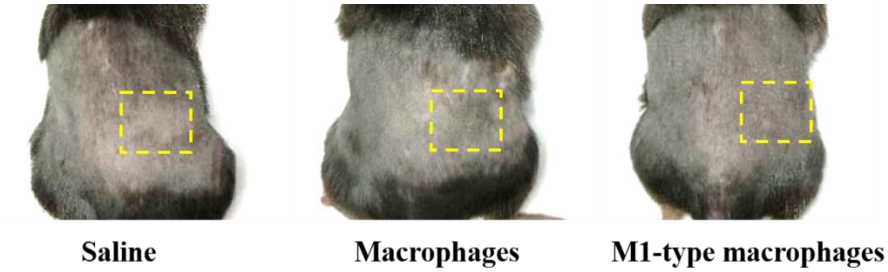

Fig. S16 Dermal sensitivity test of macrophages and M1-type macrophages on C57BL/6 mice. The mice were injected intradermally with saline, macrophages and M1-type macrophages, respectively, and the mice were observed and photographed at 24 h. The yellow area represents the intradermal injection area

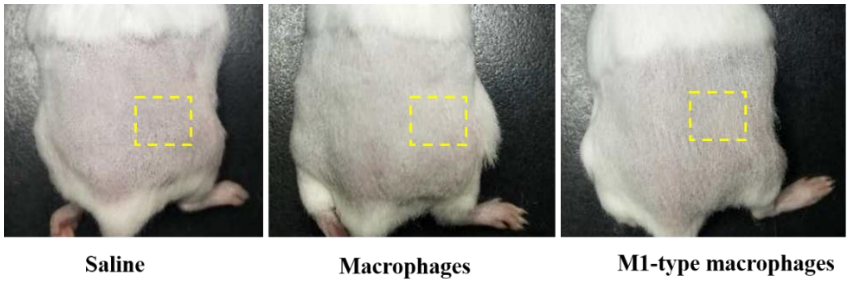

Fig. S17 Dermal sensitivity test of macrophages and M1-type macrophages on Kunming mice. The mice were injected intradermally with Saline, Macrophages and M1-type macrophages, respectively, and the mice were observed and photographed at 24 h. The yellow area represents the intradermal injection area

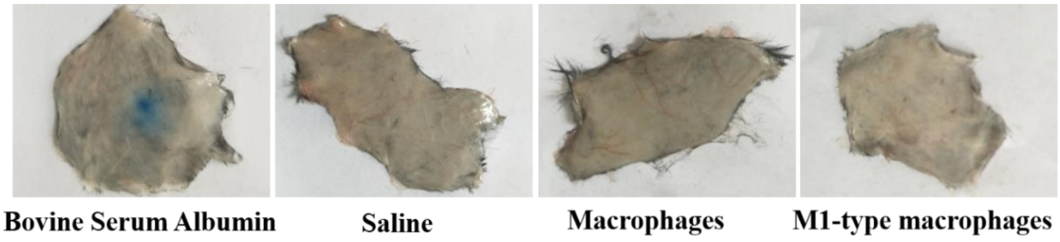

Fig. S18 Passive cutaneous anaphylaxis test of macrophages and M1-type macrophages on C57BL/6 mice

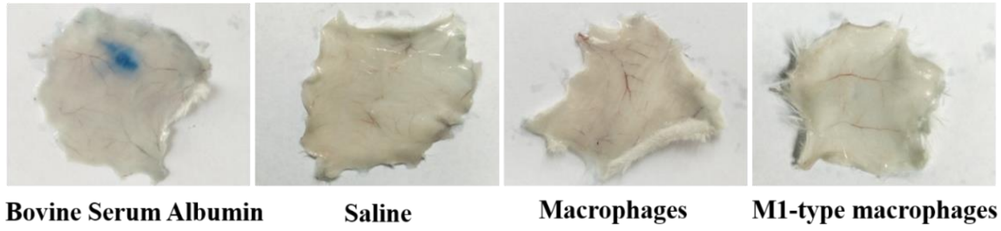

Fig. S19 Passive cutaneous anaphylaxis test of macrophages and M1-type macrophages on Kunming mice

S2 Supplementary Tables

Table S1 Characterization of SLNP

	Size (nm)	PDI	(DL%)
SLNP	67.63±5.02	0.159±0.018	5.58±0.41

Table S2 Characterization of M/SF, M/SLNP and M1/SLNP

Formulation	Drug-loading (μg/10^6cells)
M/SF	24.46±1.33
M/SLNP	37.43±0.53**
M1/SLNP	38.18±0.80**

* * $p < 0.01$, compared with M/SF

Table S3 Tumor targeting efficiency of different group at 12 h

Group	Free Cy5.5	Cy5.5-LNP	M/ Cy5.5-LNP	M1/ Cy5.5-LNP
Targeting efficiency(%)	2.57±0.18	10.00±1.46*	16.22±1.66**#	17.43±0.16**#

**p< 0.01, *p< 0.05, compared with free Cy5.5 group; #p< 0.05, compared with Cy5.5-LNP group

Table S4 Tumor targeting efficiency of different group at 24 h

Group	Free Cy5.5	Cy5.5-LNP	M/ Cy5.5-LNP	M1/ Cy5.5-LNP
Targeting efficiency(%)	8.60±2.00	13.54±1.6	22.39±1.65**##	24.12±2.57**#

**p< 0.01, compared with free Cy5.5 group; ##p< 0.01, #p< 0.05, compared with Cy5.5-LNP group

Table S5 IC_{50} values of M1/SLNP

Group	released medium of M1	released medium of M1/LNP	Free SF	SLNP	released medium of M/SLNP	released medium of M1/SLNP
IC50±SD(μg/ml)	11.74±0.12	12.33±0.89	7.62±0.37	4.62±0.55**#	5.13±0.52**##	2.4±0.23***

***p < 0.001, **p < 0.01， compared with free SF; ##p < 0.01, #p < 0.05, compared with released medium of M1/SLNP
